# Cognitive flexibility in verbal and nonverbal domains and decision making in anorexia nervosa patients: a pilot study

**DOI:** 10.1186/1471-244X-11-162

**Published:** 2011-10-07

**Authors:** Giovanni Abbate-Daga, Sara Buzzichelli, Federico Amianto, Giuseppe Rocca, Enrica Marzola, Shawn M McClintock, Secondo Fassino

**Affiliations:** 1 Eating Disorders Center, Department of Neuroscience, University of Turin, Italy; 2 University of Texas Southwestern Medical Center at Dallas, Psychiatry Dallas, Texas, USA; 3 New York State Psychiatric Institute/Columbia University, Psychiatry New York, New York, USA

## Abstract

**Background:**

This paper aimed to investigate cognitive rigidity and decision making impairments in patients diagnosed with Anorexia Nervosa Restrictive type (AN-R), assessing also verbal components.

**Methods:**

Thirty patients with AN-R were compared with thirty age-matched healthy controls (HC). All participants completed a comprehensive neuropsychological battery comprised of the Trail Making Test, Wisconsin Card Sorting Test, Hayling Sentence Completion Task, and the Iowa Gambling Task. The Beck Depression Inventory was administered to evaluate depressive symptomatology. The influence of both illness duration and neuropsychological variables was considered. Body Mass Index (BMI), years of education, and depression severity were considered as covariates in statistical analyses.

**Results:**

The AN-R group showed poorer performance on all neuropsychological tests. There was a positive correlation between illness duration and the Hayling Sentence Completion Task Net score, and number of completion answers in part B. There was a partial effect of years of education and BMI on neuropsychological test performance. Response inhibition processes and verbal fluency impairment were not associated with BMI and years of education, but were associated with depression severity.

**Conclusions:**

These data provide evidence that patients with AN-R have cognitive rigidity in both verbal and non-verbal domains. The role of the impairment on verbal domains should be considered in treatment. Further research is warranted to better understand the relationship between illness state and cognitive rigidity and impaired decision-making.

## Background

Eating disorders (ED) are complex neuropsychiatric illnesses with unpredictable outcomes [1]. Several risk factors, such as biological vulnerability, psychological, environmental, and social aspects are involved in the development of ED. There is general consensus that certain features of Anorexia Nervosa (AN) play a key-role in the pathogenesis of the illness. These features include a rigid focus on food, obsessive traits, hypo-affectivity, aggressiveness, and a perfectionistic, rigid, and inflexible personality [2-4].

From a neuropsychological standpoint, cognitive flexibility and decision making processes [5-7] were reported to be impaired in AN patients; moreover, these cognitive impairments were found to correlate with presenting clinical features (e.g., disturbance of body image) [8]. Regarding the domain of cognitive flexibility, AN patients tend to show difficulties in set shifting which may be related to other impaired neurocognitive functions (e.g., poor response inhibition) and clinical characteristics of perfectionism [3,8]. In a recent paper, Zastrow and Coworkers [9] used functional magnetic resonance imaging (fMRI) to investigate neural correlates of cognitive inflexibility in AN patients and in healthy controls. They found that the ventral anterior cingulate-striato-thalamic loop was hypoactive and the frontoparietal lobe was hyperactive during set-shifting tasks in AN patients.

Cognitive inflexibility, mental rigidity, and difficulty with decision making processes are probably all involved in the etiopathogenesis and maintenance of AN [10]. Although it could be speculated that a correlation between cognitive impairment and malnutrition in AN is possible, recent evidence suggests that neuropsychological performance is unrelated to Body Mass Index (BMI) or starvation [3,11]. Therefore, further research is warranted to determine what factors contribute to cognitive flexibility impairment in AN. Two recent meta-analyses [3,12], regarding neuropsychological performance in AN, reported that prior studies were limited by small sample sizes and not controlling for confounding factors (e.g., demographic and clinical characteristics). To date, in patients with ED there is no information regarding cognitive flexibility in verbal abilities. These skills are important in psychotherapy, the primary therapeutic approach used in the treatment of AN.

The Hayling Sentence Completion Task (HSCT) was used in this study to obtain a verbal domain measure of cognitive rigidity. The HSCT is a neuropsychological test of executive function created by Burgess and Shallice [13] and it is a useful instrument to assess if AN patients can perform tasks using both a good and flexible strategy while exerting appropriate control/inhibition of their responses. Indeed it is a measure of cognitive flexibility, response initiation, and response suppression. In the existing literature, the HSCT has been successfully used to evaluate these neuropsychological factors in patients with Parkinson's Disease [14], in children with Attention Deficit Hyperactivity Disorder [15], recently in patients with Unipolar Depression [16], and in fMRI studies on prefrontal activation in Bipolar Disorder and Schizophrenia [17].

The aim of this study was to a) replicate previous data regarding cognitive flexibility and decision-making impairment in a cohort of AN-R patients, b) better understand whether cognitive inflexibility persists in verbal tasks as measured on the HSCT, and c) examine whether differences in neuropsychological test performance are associated with years of education, BMI, disease duration, and depression severity. We hypothesized that cognitive impairment would be confirmed in AN-R patients using the HSCT, and that differences in cognitive inflexibility between AN-R and healthy controls would be independent of the above mentioned demographic and clinical variables.

## Methods

### Patients and Procedures

The sample for this study consisted of 60 participants, 30 patients diagnosed with Anorexia Nervosa Restrictive type (AN-R) and 30 age-matched healthy controls (HC). The AN-R patients were consecutively recruited from the Eating Disorders Center of the University of Turin (San Giovanni Battista Hospital). Of the 30 patients, 10 were partially hospitalised, 3 were hospitalised, and 17 were outpatients. All participants provided written informed consent according to the Ethical Committee of the Department of Neuroscience of the University of Turin.

Patients were included in this study who met the Structured Clinical Interview for DSM Disorders (SCID-I) [18] diagnostic criteria for Anorexia Nervosa Restrictive type (AN-R) throughout the prior year. The SCID-I was administered by an experienced psychiatrist. Inclusion criteria were a) no severe medical comorbidity (e.g., epilepsy or diabetes); b) no drug dependence; and c) female gender. Patients were all Caucasian. Healthy control (HC) participants were recruited from the University of Turin, and were only included in the study if they were free of psychiatric and medical illnesses.

After completion of study related assessments, all patients underwent individualized pharmacotherapy and psychotherapy. Because of illness severity and non-responsiveness to treatment, a large part of the sample (n = 12) had already received previous treatment in our ED Program.

We decided not to include the AN Binge-Purging subtype (AN-BP) because it is known that AN-BP individuals are different from AN-R patients concerning several features that could affect neuropsychological performances. In detail, personality characteristics - mainly impulsivity - [2,19], obsessive traits [20,21], prognosis [22], and set shifting [11] have been shown to be significantly different between subgroups. Moreover, recent preliminary neuroimaging data showed how AN-BP and AN-R individuals differ, particularly with regard to impulsivity [23].

More in detail, impulsivity and obsessive traits can differentiate these two subtypes on neuropsychological tests. This could represent a limitation for this study and future research; thus future investigation of AN-BP subtype is needed.

### Measures

#### Neuropsychiatric Assessment

We assessed characteristics of the eating disorder illness in the AN-R sample with the Eating Disorder Inventory-2 (EDI-2) [24] and Body Mass Index (BMI), and depression severity was evaluated with the Beck Depression Inventory (BDI) [25]. Overall functioning was evaluated with the Global Assessment of Functioning scale (GAF) [26].

#### Neuropsychological Assessment

We used a comprehensive neuropsychological battery comprised of the Wisconsin Card Sorting Test (WCST), Iowa Gambling Task, Trail Making Test Parts A and B (TMT), and the Hayling Sentence Completion Task (HCST). We employed the WCST (pen-paper version) [27,28] to assess abstraction ability and cognitive strategies in response to changing environmental contingencies. We examined, according to Laiacona and Coworkers [29] the following quantitative measures of the WCST: a) global score that represents an overall index of WCST performance and estimates how many cards the subject actually used in excess of the minimum necessary to achieve the six categories (global score = n of trials - [n of achieved categories ×10]), b) perseverative errors, c) non-perseverative errors, and d) failure to maintain set. The Iowa Gambling Task [30] measures decision-making ability as the subject is required to select advantageous or disadvantageous cards from four decks with the goal of maximising profit. On the TMT parts A and B [31], the subject connects numbers in ascending order and numbers and letters in ascending order, respectively, to provide information regarding attention and cognitive flexibilty. The HSCT [13] requires the subject to complete 15 sentences by filling in the correct missing word (part 1) and a nonsense word (part 2) and provides an index of response initiation and suppression.

### Statistical Analyses

For statistical analysis, we used the Statistical Package for Social Sciences (SPSS) (SPSS 13.0 Application Guide. Chicago: SPSS, Inc., 2004). Statistical power for the comparison of means in two independent samples was set at an alpha level < 0.05. As such, the sample provided sufficient power for the key variables of the study (e.g., WCST global score 88.4%; HCST Net score 80.4%; IGT Net score 97.2%). An independent-sample *t*-test was used to evaluate significant differences between AN-R and HC performances on neuropsychological tests. An independent-sample *t-*test was also used to evaluate if there were significant differences in severity of illness and neuropsychological performances between inpatients (hospitalised and partially hospitalised, n = 13) and outpatients (n = 17). The correlation between duration of illness and neuropsychological tests scores was evaluated by Pearson's linear correlation. To check the influence of years of education, BMI and depressive symptoms (BDI) on test performances, a univariate general linear model (UGLM) was used. We proceeded in three steps. The first step was to evaluate differences in the neuropsychological tests for years of education, the second step was to evaluate differences on neuropsychological test performance accounting for years of education and BMI together, and the third step was to add the BDI as a covariate. As the study was for hypothesis generating, we did not adjust statistical analyses for multiple comparisons.

## Results

### Clinical and demographic features of the sample

We enrolled a total of 60 participants, thirty per group, who were matched for gender (all participants were female and Caucasian) and age (AN-R = 24.13 ± 6.16 years, HC = 24.67 ± 2.64 years; see Table 1). The HC group differed from the AN-R group with respect to years of education (15.53 ± 2.64 versus 12.37 ± 2.82 years, respectively, F = 0.015, *p *= 0.01), which was controlled for in subsequent analyses as a confounding variable. The AN-R group differed from the HC group on all of the EDI-2 subscales, had a lower BMI, and demonstrated higher depression severity on the BDI. The GAF scores for the AN-R group were all higher than 50 while those for the HC group were all higher than 90.

**Table 1:** Demographic and clinical characteristics of the sample.

		AN-R (N = 30)	HC (N = 30)	F	*P *(t-test)
Age		24.13 (±6.16)	24.67 (±2.64)	16.66	0.66
Years of education		12.37 (±2.82)	15.53 (±2.64)	0.01	0.01
BMI		15.62 (±1.66)	21.04 (±2.18)	1.67	0.01

Age at onset		18.12 (±3.33)	-	-	-
Duration of illness		5.20 (±4.19)	-	-	-
BDI		11.77 (±7.85)	1.83 (±1.95)	34.18	0.01
EDI-2	DT	11.69 (±7.16)	1.03 (±2.08)	38.96	0.01
	B	2.07 (±2.40)	0.50 (±1.12)	12.47	0.01
	BD	11.07 (±5.88)	5.20 (±4.44)	1.96	0.01
	I	10.03 (±7.98)	1.70 (±3.44)	24.11	0.01
	P	5.00 (±3.31)	2.67 (±3.13)	0.60	0.01
	ID	5.83 (±4.71)	1.17 (±1.97)	21.17	0.01
	IA	7.52 (±5.92)	0.73 (±1.26)	43.12	0.01
	MF	6.28 (±4.53)	2.33 (±2.43)	6.25	0.01
	A	6.03 (±5.11)	2.63 (±2.34)	7.34	0.01
	IR	4.24 (±4.67)	0.63 (±1.16)	37.20	0.01
	SI	7.55 (±5.21)	2.03 (±2.98)	10.71	0.01

Female AN-R patients and healthy controls.

Mean and (standard deviation) are provided.

Statistically significant differences are shown in bold under *p *values.

BMI: Body Mass Index

BDI: Beck Depression Inventory

EDI-2: Eating Disorder Inventory-2

DT: drive for thinness,

B: bulimia,

BD: body dissatisfaction,

I: ineffectiveness,

P: perfectionism,

ID: interpersonal distrust,

IA: interoceptive awareness,

MF: maturity fears,

A: asceticism,

IR: impulse regulation,

SI: social insecurity.

No significant differences were found between the inpatient and outpatient cohorts with regard to weight (BMI = 15.51 ± 2.07 and 15.71 ± 1.33, respectively), EDI-2 scores, and the majority of neuropsychological tests (*p *> 0.05). However, relative to the outpatient cohort, the inpatient cohort showed worse performance on the Iowa Gambling Task (*p *= 0.01).

The Hayling Sentence Completion Task performance was partially correlated with duration of illness in the AN group. In particular, there was a significant positive correlation between duration of illness and HSCT net score (Pearson Correlation r = 0.379, *p *= 0.039) and the number of completion answers (Pearson Correlation r = 0.451, *p *= 0.012). The higher the duration of illness, the worse is the performance of cognitive flexibility on the HSCT. With regard to other neuropsychological tests and BMI, no significant correlations were found with duration of illness.

### Cognitive Flexibility Function

No significant differences were found between the AN-R and HC groups on the TMT Parts A and B (*p *> 0.05). However, the other neurocognitive tests measuring cognitive flexibility showed significant differences between groups. Specifically, the AN-R patients' mean performance on the WCST was significantly worse than that of HC. Both global score and number of errors (perseverative and non-perseverative) were significantly higher in AN-R patients (Table 2). When we corrected differences between groups for years of education, all differences, with the exception of perseverative errors, remained significant. When we corrected analyses for BMI, the differences between the groups on the WCST were found to be not significant. Finally, when we controlled for the BDI, the differences in WCST scores were no longer significant.

**Table 2:** Results of neuropsychological assessment.

		AN-R (N = 30)	HC (N = 30)	F	*P *(t-test)	*P *(UGLM)
WCST	Global score	29.40 (±28.66)	12.83 (±3.42)	21.66	**0.01**	0.94*
	Perseverative errors	8.57 (±7.9)	4.97 (±1.01)	17.13	**0.01**	0.85
	No-Perseverative errors	9.47 (±8.98)	4.07 (±1.96)	15.33	**0.01**	0.83
	Failure	0.73 (±1.89)	0.03 (±0.18)	9.55	**0.04**	0.99

HSCT	Net Score	3.73 (±2.64)	2.23 (±1.36)	18.47	**0.01**	0.11
	Answer C	0.13 (±0.35)	0.00	24.93	**0.03**	0.27§
	Answer S	3.33 (±2.25)	2.23 (±1.36)	8.96	**0.02**	**0.03**
	Answer U	6.53 (±2.34)	7.77 (±1.36)	13.42	**0.01**	**0.05**

IGT	Net Score	-3.60 (±19.44)	16.13 (±20.22)	0.48	**0.01**	0.13
	First Fifty	-5.60 (±12.39)	7.27 (±11.82)	0.12	**0.01**	**0.01**
	Second Fifty	2.00 (±11.00)	11.47 (±14.5)	1.09	**0.01**	0.48

Female AN-R patients and healthy controls.

Mean and (standard deviation) are provided.

*P *(UGML): significance corrected for years of education. BMI. and BDI scores.

*: BMI was significantly associated with the test score.

§: BDI was significantly associated with the test score.

Statistically significant differences are shown in bold under *p *values.

WCST: Wisconsin Card Sorting Test,

HSCT: Hayling Sentence Completion Task,

IGT: Iowa Gambling Task,

Answer C: sentence completion,

Answer S: semantic-related answer,

Answer U: semantic-unrelated answer.

The differences between AN-R and HC groups performance on the HSCT were significant (Table 2), with scores higher in the AN-R group. No significant differences between groups were found in the task performance times. The differences between AN-R and HC groups on the HSCT, when corrected for years of education, remained significant (*p *< 0.05). After controlling for BMI, the HSCT scores, except for the Hayling C score (*p *= 0.427), remained significant. Finally, when we controlled for the BDI, only the Hayling S and U scores remained significant (*p *< 0.05).

### Decision Making Function

Results showed that the IGT Net and sub-scores were significantly lower (*p *< 0.05) for AN-R patients relative to the HC participants (Table 2). When analyses were corrected for years of education, the differences in IGT scores between the AN-R and HC groups remained significant (*p *< 0.05). When BMI was controlled, all IGT scores remained significant, except for the score on the second 50 (out of 100) choices (*p *= 0.182). With all three confounding variables (years of education, BMI, and BDI) controlled, only the score for the first 50 choices (out of 100) remained significant (*p *< 0.05).

## Discussion

This study confirms previous literature showing that patients with Anorexia Nervosa have difficulty with cognitive flexibility and decision making processes [7,10,32-35].

Decision making ability, as shown in a recent article [36], seems to be partially related to the state of the illness (partially or fully hospitalized patients in the acute phase of illness performed worse at the neuropsychological tests than the outpatients), while cognitive flexibility in this study is confirmed to be independent from the phase of illness.

Duration of illness was found to be unrelated to performance on most of the neuropsychological measures, with the exception of the HSCT. Specifically, more errors on HSCT Part B were associated with a greater duration of illness. This is clinically meaningful as the errors are suggestive of an inability to inhibit automatic responses, which could have implications for psychotherapeutic interventions. About HSCT, it is possible indeed that long standing patients show higher cognitive impairments in set shifting, given their main use of verbal and cognitive automatisms.

Regarding education, there was no relationship with neuropsychological impairment, suggesting that it does not act as a protective factor in this patient population. On the other hand, it is well-known that the IQ of these patients is higher than average [37] and it is not lowered by their low weight: these data seem to suggest that less years of education can be due to the illness and not to impaired intellective abilities.

However, neuropsychological function may be related to Body Mass Index, as performance on the WCST was impacted by BMI. In previous literature, most studies did not control for BMI [10,35]. To our knowledge, only Mathias and Kent [38] reported a correlation between TMT Part B and BMI, while other authors reported no correlations with WCST performance [8,32,33]. Therefore, our data are not in line with previous investigations about this topic. It might be possible to explain this by examining the characteristics of our sample. The sample indeed was clinically heterogeneous if we consider that we recruited only patients who were both inpatients (partially hospitalised and in-hospitalised) and outpatients. These features entail a BMI range different from the majority of participants recruited in prior investigations [7,32,33,39]. Because the study is cross-sectional, these data suggest that future longitudinal researches are warranted to address this issue. Theoretically, the association between BMI and performance on the WCST is difficult to explain in a cross-sectional study. Low BMI is intertwined with AN-R, which limits the ability to distinguish between the two factors to determine how each independently impacts problem solving abilities. One study [40] found that AN patients who recovered still showed poor set shifting abilities, and another study of 215 AN patients found no relationship between BMI and a problem solving task using the Brixton test [41]. Thus, future research is needed to investigate the impact of weight on neuropsychological performance with an examination of other indices besides the BMI.

The HSCT, used for the first time in this context with AN-R patients, showed interesting results. The AN-R patients showed an inability to inhibit spontaneous answers and to establish a flexible strategy as required for a good test performance. Not only was the general score significantly worse for AN-R patients, but also the number of errors on all sub-scores was greater compared to the HC group. AN-R patients provided significantly more semantic-related answers in the second part of the test, indicating a lack of good strategy formation. No differences were observed in thinking time, probably because the difficulties of AN-R patients are unrelated to a lower processing speed [8]. On the HSCT, we found that cognitive inflexibility characterising AN-R patients was linked with both verbal and non-verbal domains. To our knowledge, this is the first study to show poor cognitive flexibility in both verbal and nonverbal domains of patients with AN-R.

Impaired performance on the HSCT has been found in other psychiatric illnesses such as Schizophrenia [42,43], Bipolar Depression [44,45], and Major Depressive Disorder [16]. Nevertheless, the response inhibition process and the verbal fluency impairment in our sample seemed to be independent of depressive symptomatology. Our hypothesis is that other mechanisms not involving processing speed and attentional deficits related to depression could underscore the lower performances of AN-R patients. Thus, it is possible that the cognitive deficits found in this study may be related to obsessive traits typical of AN-R [5,46]. In fact, prior research has shown that patients with Obsessive-Compulsive Disorder (OCD) perform worse than HC and phobic patients on the HSCT [47]. Future studies addressing cognitive flexibility in verbal and nonverbal domains should compare ED patients with other psychiatric cohorts - mainly OCD patients - to examine this possibility.

Regarding performance on the IGT, AN-R patients performed significantly worse than the HC group suggesting they have impaired decision-making strategies. This finding was unrelated to depression severity. AN-R patients tended to make disadvantageous choices for the first 50 trials (i.e., the amount of money won each time was higher, but they did not consider that the amount they were losing was even bigger) and showed a preference to choose to risk without realising that they were choosing a fictitious, even if immediate, advantage instead of a long-term, but real one. These data confirm the cognitive inflexibility in AN-R patients and the difficulty they have to inhibit incorrect answers, as shown in this study by the HSCT results. On the second part of the test, this difference did not remain after controlling for confounding variable (BMI) and, also in this case, the performance was not totally independent of weight.

Considering all the neuropsychological tests used, cognitive inflexibility and decision-making impairment seemed to be only partially independent of the state of the illness. BMI effect may be correlated to malnutrition but also to psychopathology and to state of the illness, as discussed above. Depressive symptomatology seemed to be associated to some extent with the performance differences found between the groups. It is well-known in literature that a subgroup of AN patients shows comorbid Major Depression [48]. Even though the effects of depression on neurocognitive functions are heterogeneous and still poorly understood [49], depression in AN-R individuals could affect cognitive flexibility, especially involving component processes (e.g., attention) [50] and serotonin dysregulation [51].

In our study, cognitive inflexibility and decision-making impairments in AN-R could be biological markers of the illness. According to previous literature, a biomarker can be defined as a measurable indicator of disease that is modified by the course of illness [34,52]. Because this study provides evidence that neuropsychological features are not completely state-independent, it is important that longitudinal studies assess the extent that cognitive flexibility may actually be an endophenotype of AN-R [11]. Both cognitive flexibility, which can also be investigated with a verbal test like the HSCT, and impairments in decision-making strategy are considered characteristics of AN-R patients. These traits could probably play a role in the enhancement and maintenance of the disorder, at least for this subtype of AN patients [10,53,54].

This study involved a large sample of patients diagnosed with restrictive type of Anorexia Nervosa, and a new verbal test (HCST) to evaluate cognitive flexibility. Since we included both outpatients and inpatients, our study sample was heterogeneous with regard to BMI and severity of illness. Moreover, with this sample we provided further data about alterations previously reported in literature in more severe AN-R patients [7,10]. The main limitation of this study was the lack of a clinical control group, such as a group of patients affected by AN-BP, Bulimia Nervosa, or OCD, to better evaluate the role of cognitive flexibility and decision-making abilities across different diagnostic categories. This would have allowed us to address the question if these impairments are specific to AN-R, or if they are characteristic of eating disorders or other psychiatric pathology in general. Moreover, we did not evaluate for anxious symptomatology, which could have impacted neurocognitive performance. While prior research suggests that anxiety may not negatively affect set shifting abilities [11], future research should include measures of anxiety symptoms to better clarify the effects, if any, on cognitive flexibility in patients with AN-R.

Also, we did not adjust our statistical analyses for multiple comparisons, which could have resulted in false-positive findings. However, our study was intended to be hypothesis generating and using a statistical correction (e.g., Bonferroni) could have been too conservative. Future studies are warranted to confirm these results and to collect longitudinal and family data. From a clinical standpoint, it would be useful to develop a specific cognitive remediation therapeutic approach based on the neuropsychological findings [35,55] that is focused on the improvement of cognitive flexibility and decision making processes in AN-R patients.

## Conclusions

This study provided evidence that cognitive flexibility and decision-making strategies are impaired in patients with Anorexia Nervosa. Moreover, we found that cognitive rigidity is linked not only with non-verbal but also with the verbal domain. These data raise new possibilities for future research in this field. The second aim of this study was to evaluate the role of BMI in neuropsychological performances and we confirmed that a linear correlation was not reported, but that BMI could play a role to some extent in cognitive flexibility and decision making impairment in AN patients.

## Competing interests

The authors declare that they have no competing interests.

## Authors' contributions

GAD was the major contributor to the writing of this paper; SB collected all data; GR did the statistical elaboration of the data; EM reviewed the paper and translated it, SMM reviewed the paper and translated it, FA and SF contributed to essential aspect of the paper and reviewed it extensively.

All authors read and approved the final manuscript.

## Pre-publication history

The pre-publication history for this paper can be accessed here:

http://www.biomedcentral.com/1471-244X/11/162/prepub
